# Hybrid barn: the switch from a naturally to a mechanically ventilated turkey barn to protect from harmful bioaerosols

**DOI:** 10.3389/fvets.2025.1443139

**Published:** 2025-02-19

**Authors:** Björn Sake, Kira Butenholz, Katrin Kempf, Nicole Kemper, Jochen Schulz

**Affiliations:** Institute for Animal Hygiene, Animal Welfare and Farm Animal Behavior, University of Veterinary Medicine Hannover, Foundation, Hannover, Germany

**Keywords:** airborne disease, air filtration, biosecurity, animal hygiene, avian influenza, animal health, animal welfare, bioaersols

## Abstract

**Introduction:**

Animal health is essential to ensure the highest level of animal welfare and to conserve resources. Especially in naturally ventilated barns, the airborne entry of pathogens is difficult to avoid. For instance, birds in naturally ventilated turkey barns are frequently infected by highly pathogenic avian influenza and the airborne route may play a role. In this study, a naturally ventilated turkey barn was equipped with filter modules that allow a conversion to a mechanically ventilated barn.

**Methods:**

Four filter modules with two filter stages were adapted to a turkey barn and the curtains were closed to induce a slight overpressure in the barn. Air samples were taken over the course of 16 months in front of and behind the filter units to assess the filter efficiencies. Filter efficiencies were evaluated by the reduction of particle classes (PM1, PM10, nanoparticles), microorganisms (total bacteria, molds, yeasts), and the detection of potentially pathogenic bacteria and viruses by PCR tests.

**Results:**

Particle reduction rates were 94.17% for PM1, 94.27% for PM10, and 95.80% for nanoparticles, respectively. Total bacteria counts were reduced by 95.88%, molds by 94.64%, and yeasts by 66.03%. *Ornithobacterium rhinotracheale* was significantly retained in the filter units. The results for influenza A also indicated that the entry of potentially infectious particles could be prevented.

**Discussion:**

The flexible switch from a naturally to a mechanically ventilated barn with filtered supply air can be an innovative solution to avoid airborne pathogen entry in risky situations and may represent a component in the strategy to control epidemic diseases.

## 1 Introduction

Keeping livestock healthy and thus maintaining productivity is one of the major challenges in modern livestock farming. Infectious diseases caused by microorganisms and viruses continue to play an important role. The routes of entry into a barn are manifold. Among the various modes of transmission, airborne routes have garnered considerable attention due to their potential impact on disease spread within livestock facilities (1, 2). Airborne diseases are transmitted by aerosols, which are liquid or solid particles suspended in the air that act as carriers for infectious agents (3). These pathogens, termed airborne pathogens, are released into the environment by infected animals through particles of potentially infectious secretions and excretions. Ingestion or inhalation of these particles by animals can result in infection (2, 4). Once airborne, pathogens can disperse with air currents, remain suspended, or settle on surfaces. It is difficult to distinguish between true airborne transmission and transmission from air and contaminated surfaces (1). Potentially infectious particles agglomerate with dust particles or are attached to feather components and skin cells. These are termed bioaerosols, containing both living and active (microorganisms, viruses) and inanimate (dust) components as well as complex particles, which may exhibit increased resistance to environmental influences such as radiation, dehydration, or oxidation. This resistance, known as tenacity, allows microorganisms and viruses to remain infectious over long distances and long times (5, 6). Several factors, including strain genetics, aerosol characteristics, duration and concentration of shedding, and environmental conditions, influence airborne transmission (4). As a result, it is difficult to predict or retrospectively detect airborne transmission. However, it seems evident that airborne transmission can play a role in inter-farm spread, particularly in densely populated areas (1, 7–9). For instance, Dee, Otake et al. showed that infectious porcine reproductive and respiratory syndrome viruses (PRRSV) were transmitted via the airborne route over a distance of 120 m and infected recipients, housed in a non-filtered building (10). Further, beside the distance and the virus concentrations, the type of poultry house seems to play a crucial role in the probability of airborne infection. Nguyen, Zhao et al. calculated that turkey farms showed the highest chance of being infected by long-distance airborne transmission of AI (11). The authors argued, that next to the susceptibility the open side walls and the higher ventilation rates in turkey house increases the chance to get airborne AI transmitted from infected farms. Understanding the complexity of this kind of transmission is crucial for the implementation of effective preventive measures, particularly to mitigate the spread of disease in livestock.

In poultry, one example of such a complexity is the transmission of avian influenza (AI). Avian influenza viruses are divided into different subtypes. They are classified into highly pathogen (HPAI) and low pathogen (LPAI) subtypes based on their pathogenicity. The pathogenicity of these viruses depends on the properties of hemagglutinin, which binds to neuraminic acid on the target cells. The subtypes H5 and H7 belong to the HPAI and the colloquial term of the disease is avian flu. In recent years, AI has repeatedly caused high economic losses in commercial poultry farming (12). The viruses are spread by secretions and feces of wild birds or via contaminated material. Despite increased biosecurity and sensitization of farmers, repeated infections may occur. Since AI appears to be able to spread from farm to farm, the risk could increase in areas with high poultry densities (7). To protect poultry from infection, it is therefore important to maintain accurate biosecurity and to avoid contact to potential sources from outside. For example, contact with wild birds or with contaminated fomites and uncontrolled access of people must be avoided as far as possible. Careful use of the hygiene lock, including changing of clothes and shoes, and good hand hygiene also reduce the risk of introducing infectious agents. Despite careful hygiene measures, it is not always possible to prevent entry. In general, airborne transmission, e.g., through contaminated fecal particles, droplets, dust or feathers is considered to play only a minor role for AI (8). However, the importance of the airborne entry is constantly being debated, although there are indications that pathogens can be transmitted from flock to flock by air through aerosols directly or from deposited particles under certain conditions (2, 7, 11–17). The airborne entry represents an uncontrollable path of entry. For instance, if farms in one region are infected, birds might be killed for reasons of disease control, posing a potential risk to neighboring farms. The relevance of the airborne entry differs for the various outbreaks. However, depending on the conditions, this cause can be in the double-digit percentage range (8, 11). In particular, naturally ventilated barns with open sidewalls are especially susceptible to airborne entry (11). Fattening turkeys are mostly kept in open, naturally ventilated barns (18), which are therefore potentially susceptible to aerosols and airborne infections. Particularly turkey barns, which must ensure a high level of air exchange, have been affected in past AI outbreaks. In these types of barns, the incoming air cannot be filtered because the air resistance of the filters would inhibit necessary air rates. However, filtering the supply air may reduce the risk of potentially infectious particles entering the air (19). A solution might be a conversion from a naturally ventilated to a mechanically ventilated barn with a filter system.

In this study, a naturally ventilated turkey barn was modified to a hybrid barn that allows the switch from naturally to mechanically ventilation and back. When mechanically ventilation is turned on, cooling pads in the filter modules can also cool the supply air to avoid heat stress. The advantage of the systems is that it is still an open barn that can be run cost effectively with naturally ventilation but offers the possibility of maintaining a good and healthy barn climate in hazardous situations or temperature inversion. The system can be retrofitted to existing barns and provide the animals with sufficient quantities of filtered fresh air. During this phase, the curtains remain closed, and the incoming air creates a slight overpressure in the barn. This is designed to prevent virus-laden particles from outside entering the barn interior through the remaining openings and thus improve biosecurity. The aim of the research project was to evaluate these filter modules considering their potential to reduce particles and bioaerosols in the supplied air. Furthermore, climate parameters were measured during the farm visits to estimate the air quality and the distribution of fresh air in the barn. Up to now, scientific evidence for the applicability of such filter modules in the agricultural environment under practical conditions are not available yet.

## 2 Materials and methods

### 2.1 Animals and housing

One naturally ventilated turkey barn (15 × 44m) in a densely populated turkey area in North West Germany was selected for modification and investigation. The farm was a fattening farm, where tom turkeys were housed after a five-week rearing period and were fattened until 21 weeks of age. The barn housed 1,700 tom turkeys (B.U.T. 6, Aviagen Turkeys Ltd., Cheshire, United Kingdom), and the maximum stocking density was 58 kg/m^2^. When naturally ventilated, supply air entered through the openings on the long sides of the barn, was mixed with the inner air, warmed up and risen to be extracted through the ridge openings. The degree to which the curtains were opened at the long sides could be used to regulate the supply air. This study was reviewed and received approval from the Animal Welfare Officer of the University of Veterinary Medicine Hannover, Foundation, Hannover, Germany (TVO-2023-V-33).

### 2.2 Filter modules and adaption

**Figure 1:**
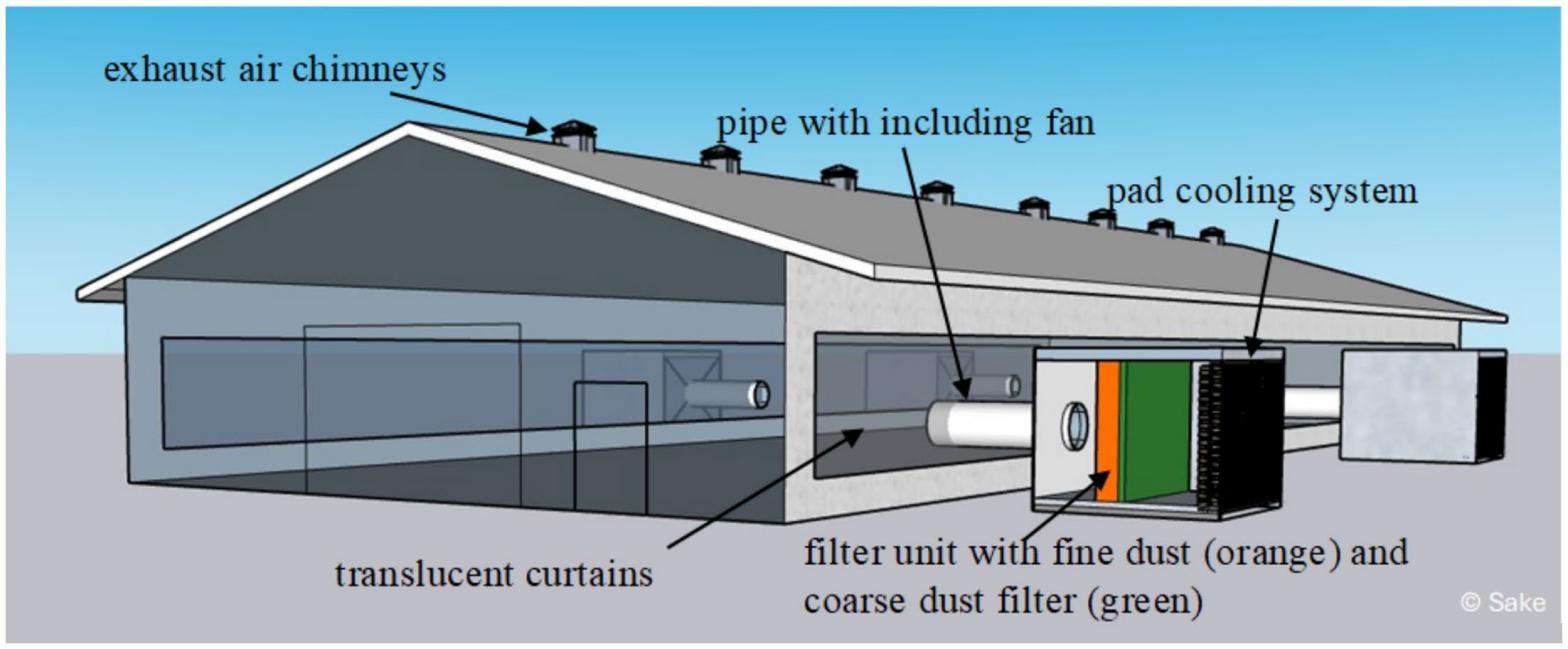
Filter modules adapted to the turkey barn were connected to the barn via large pipes including fans. The air passed through the pad cooling system and a filter unit before being pressed into the barn. The translucent curtains at the sidewalls were completely closed to prevent the uncontrolled entry of particles. Created by Björn Sake.

Filter modules were developed to convert this naturally ventilated barn into a so-called hybrid barn. Each module was equipped with a large fan (up to 22,000 m^3^ air/h) and consisted of sandwich panels. These fans drew air through a filter unit and delivered it through connected pipes and valves into the barn where the air was distributed by other fans. The upstream filter unit was equipped with a coarse dust filter [Vorfilter 592x592x48 Coarse 65% (G4)] and a fine dust filter [Feinstaubfilter 592x592x296 ePM1 80% (F9)]. The filter unit divided the interior of the filter modules into two compartments of approximately equal size. Each filter unit included filter cassettes with the corresponding installation frames with spring plates to hold the filters in place. The resulting partition wall consisted of an area of 4 × 4 installation frames with the filter cassettes installed in them. The interior in front of and behind the filter unit was accessible from the outside through a small lockable door. Electrical connections for operating the measurement technology were considered during planning and implemented. Pad cooling systems were additionally installed in the filter modules. These were attached to the outer air side of the module and were operated with water. Figure 1 shows the schematic drawing of the attached modules.

Instead of supplying air along the whole of the long side, four points were selected at which a filter module was adapted. These points were also located on the long sides of the barn. Two filter modules were located in the middle opposite, two on the long sides of the barn, and the other two were installed diagonally opposite each other on the barn to ensure a good distribution of the supply air (Figure 1). For the test period (December 2022 to March 2024), the barn was permanently supplied with fresh air via the filter modules and the curtains remained closed. The curtains were translucent so that sufficient daylight continued to enter the barn. Connection points were installed inside the curtains with a wooden construction for the modules. The connection pipes that connected the filter module to the barn also contained large fans that drew fresh air through the filter unit and into the barn. The fans were controlled by the barn computer and the installed barn climate sensors. For the investigations, the curtains had to always remain closed in order to be able to investigate the filter life.

### 2.3 Data collection

Air sampling was carried out during four consecutive fattening periods (period A-D). The barn was visited at 14-day intervals, except for the first two measurements in period four (D), where the interval was only one week. Samples were taken at seven days in the first fattening period A and at eight days in each of the following three periods B-D (*n* = 31). On the sampling days, the four filter modules were sampled in random rotation. The particle measurements of the very first sampling day were removed from the results because of incomparable sampling intervals.

#### 2.3.1 Particle measurements

Two different types of devices were used to record the number of particles and their size. Two aerosol spectrometers (Dust Monitor, Model No.: 1.108, Grimm Aerosol Technik GmbH, Ainring, Germany) were used to measure particles in spectra between 0.3 and 20 μm. For the investigations, the spectra were analyzed, meaning that the numbers of particles with a diameter of up to 1 μm (particles between 0.3 and 1 μm) or up to 10 μm (particles between 0.3 and 10 μm) were considered. The devices were mounted on tripods and placed in front or behind of the filter unit in the module. Additionally, a nanoparticle counter (P-Trak® Ultrafine Particle Counter Model 8,525, TSI Incorporated, Shoreview, MN, USA) was mounted on a third tripod in front of the filter unit. This counter detected particles between 0.02 and 1 μm. After the instruments had been switched on, the measurement protocol specified an analyzable measurement time of 30 min. After that, the nanoparticle counter was placed on a tripod behind the filter unit and switched on for the comparative measurement, also of 30 analyzable minutes. The measured values were recorded and stored every 6 s. Arithmetic means and standard deviations were calculated with Microsoft Excel 2016 (Microsoft Corporation, One Microsoft Way, Redmond, WA, USA) for PM1, PM10, and nanoparticles in front of and behind the filter unit.

#### 2.3.2 Bioaerosol measurements

Bioaerosol measurements were performed simultaneously to the particle measurements. For the microbiological analyses, a Coriolis air sampler (Coriolis® *μ* Air Sampler, Bertin Technologies SAS, Montigny-le-Bretonneux, France) was used. This device separated particles in a buffer liquid using centrifugal forces. A phosphate buffered saline buffer solution was used as the buffer fluid. The Coriolis air sampler was operated for five minutes per sample and set at 200 liters per minute. To avoid contamination from the opened filter module, the start was delayed by one minute after closing the door. On each sampling day (*n* = 31), three samples were taken in front of the filter unit and three samples behind it. Three samples were also taken from the barn air for comparison. These sample liquids were then taken to the laboratory and analyzed within 24 h.

In the laboratory, the samples were analyzed for total bacterial count (TBC). For this purpose, triplets were plated on TSA (Tryptone Soy Agar, Oxoid, CM0131, Basingstoke, United Kingdom) and incubated at 37° C for 48 h. DG18 (Dichloran glycerol agar, Oxoid, CM0729, Basingstoke, United Kingdom) was used according to manufacturer to test for molds and yeasts. These plates were incubated at 25° C for a total of seven days and counted. Colony-forming units (CFU) per mL were calculated by means of the weighed mean method. Concentration of airborne microorganisms was calculated by equation (1) according to Ahmed et al. (20) and a daily arithmetic mean was calculated. Separation rates and medians for each period were calculated. For samples without any growth on all three plates, a conservative detection limit of 0.9 germs on these three plates was assumed (resulting in 7–9 CFU/m^3^). In addition, a transport control (sampling cone filled with buffer) was analyzed after each sampling day as a negative control for contamination. Some samples were later subjected to PCR analysis.

#### 2.3.3 Polymerase chain reaction

In addition to particle measurements and cultivation of microorganisms, polymerase chain reaction (PCR) was carried out to assess the reduction potential of the system. For virus detection, PCR for influenza A (IA) was included. To consider other infectious agents that could serve as indicator pathogens, the detection of two pathogens that are specifically airborne in turkeys were also analyzed. These were the avian metapneumovirus, which is responsible for Turkey Rhinotracheitis (TRT), and the *Ornithobacterium rhinotracheale* (ORT), which causes Ornithobacteriosis.

Aliquots (*n* = 56) from 15 sampling days from the Coriolis air sampler were analyzed for the occurrence of IA, ORT, and TRT. These analyzed samples derived from the first two fattening periods (A, B). It was ensured that the corresponding samples were analyzed behind the filter unit in case of positive findings in front of the filter unit.

In addition to the Coriolis air sampler, a stationary sampling point was included from the beginning of the second period (B). A total of 20 measurement periods, lasting 14 days each, were sampled between farm visits. For this purpose, a total dust sampling system (GSP10 Probenahmekopf, GSA Messgerätebau GmbH, Ratingen, Germany) was used. These GSP heads were installed once behind the filter unit in a filter module and directly next to the same filter module in the outside air on tripods. The GSP heads had been previously filled with Teflon filters (TF-1000 1.0 μm 37 mm PTFE with pads 100/pk, Pall Corporation, Ann Arbor, MI, USA) in the laboratory and were operated by vacuum pumps (Constant Flow Samplers, BRAVO BASIC R, TCR TECORA Pollution Check, Cogliate, Italy and Air sampling device for asbestos, Model X-XA21-2-2 R, Kytola Instruments Oy, Muurame, Finland). The flow rates were adjusted by rotameters of the pumps to 7.5 L/min. The flow rates were controlled at the beginning and at the end of each measurement period. After two weeks of continuous operation, the GSP heads were replaced and transported back to the laboratory well sealed in parafilm for PCR-analysis. After the four periods (A-D), dust cake in all four filter modules was collected from the coarse dust filters in front of the filter unit. These samples for PCR-analyses were washed in a 15 mL conical bottom centrifuge tube with 10 mL TE (Tris-EDTA) buffer solution and then frozen at −20°C.

The Kylt® Influenza A-PCR FLI-B (Kylt, Höltinghausen, Germany) was used for the Influenza A-PCR. The kit used for TRT diagnosis was Kylt® aMPV-A&B (Kylt, Höltinghausen, Germany). The Kylt® ORT-PCR (Kylt, Höltinghausen, Germany) kit was used for detecting ORT. A result with a CT value of less than or equal to 42 was considered positive. Positive findings were sequenced. PCR analyses were outsourced to external laboratories, namely the PHW central laboratory (PHW-Zentrallbor, Visbek, Germany) and SAN VET (SAN Group Biotech Germany GmbH, Höltinghausen, Germany), and the sequencing was performed at LAVES (Niedersächisches Landesamt für Verbraucherschutz und Lebensmittelsicherheit, Hannover, Germany).

#### 2.3.4 Additional PCR investigations

Shortly after the end of the study period, a few days before the turkeys were to be sent to the slaughterhouse after the fourth period, an acute influenza outbreak occurred in the nearby barns. PC filters (Cyclopore track etched Membrane, CYYLPR PC BLK 25 mm 0.2 μm, Whatman, Little Chalfont, United Kingdom) were inserted in the IOM Sampler (IOM Inhalable Samplers and Cassettes, plastic, SKC, Inc., Eighty Four, PA, United States) and connected to vacuum pumps and placed in front of and behind the filter unit. The pumps were set at a flow rate of 5 L/min and installed there for a total of four days. Three air samples were collected in front of and three behind the filter unit. The filters were washed in 5 mL TE buffer and stored at −20°C. These samples were analyzed only for IA as described above.

#### 2.3.5 Barn climate parameters

Barn climate data were recorded and controlled during each farm visits with hand-held instruments at nine sampling points evenly distributed throughout the barn. The following parameters were recorded: temperature [°C], relative humidity [%], air velocity [m/s], CO_2_ [ppm], NH_3_ [ppm], and light intensity [lx]. The mobile gas measurements were carried out with a gas sensor (Dräger X-am® 5,600, Drägerwerk AG & Co. KGaA, Lübeck, Germany). For the other barn climate parameters, a climate measurement sensor (testo 440 dP, Testo SE & Co. KGaA, Titisee-Neustadt, Germany) with appropriate probes was used.

### 2.4 Statistical analyses

Data processing and statistical analyses were performed using Microsoft Excel 2016 (Microsoft Corporation, One Microsoft Way, Redmond, WA, USA) and the commercial software SAS 9.4 (SAS Institute Inc., Cary, NC, USA), respectively.

The bioaerosol data were tested for normal distribution with the procedure UNIVARIATE. As there was no normal distribution, a pairwise comparison of the concentrations in front of and behind the filter unit was carried out using the Wilcoxon Rank Sum Test. The Kendall’s Tau-b correlation coefficient was calculated for the parameters nanoparticles, PM1, PM10, TBC, molds and yeasts in front of and behind the filter unit. The Fisher’s exact test was calculated for the Coriolis air sampler results of ORT. The level of statistical significance was set at *p* < 0.05.

**Figure 2:**
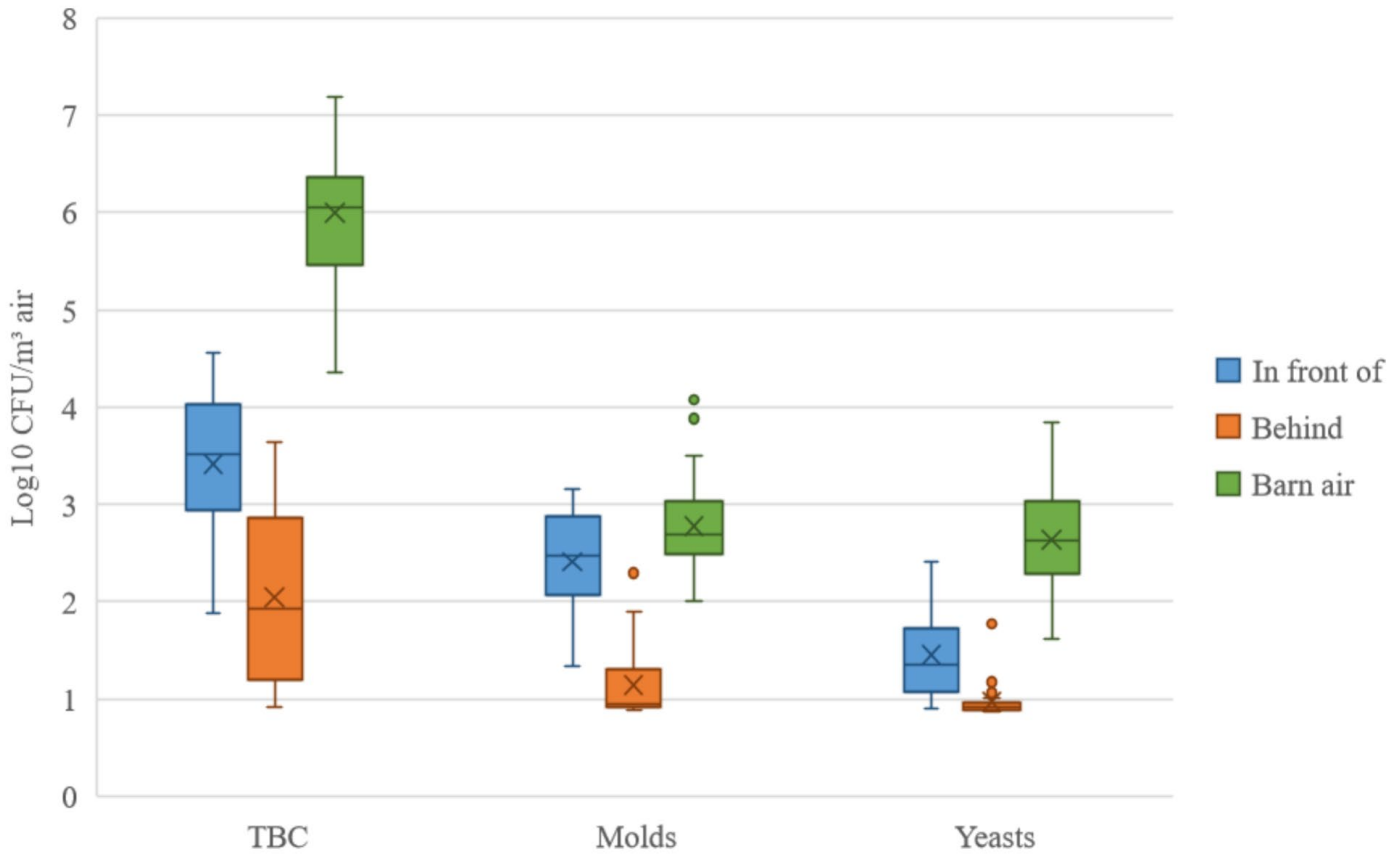
The microbiological results of the total bacteria count (TBC), molds, and yeasts. Samples were taken in front of and behind the filter unit. The medians (─), arithmetic means (X), interquartile ranges (┬ ┴) and outliers (•) are given for comparison. The values were logarithmized and given in colony-forming units (CFU) per m^3^ of air.

The arithmetic means for the barn climate parameters for the nine measurement points and in general, inclusive standard deviation, were calculated. In addition to arithmetic means, the maximum and minimum values measured were determined. To estimate the distribution of air in the barn, network diagrams were drawn with Excel Figure 2. For a better overview, the values of each point were logarithmized and displayed for each sampling point.

## 3 Results

### 3.1 Particle counting

Clear reduction rates were shown for fine dust particle classes (Tables 1, 2). The reduction rates of PM1 and PM10 ranged from 92.40 to 96.46%. The average value for all periods was 94.17% for PM1 and 94.27% for PM10 (Table 1). The reduction rates increased from period to period for PM10. The situation was similar for PM1, but there was an exception from period B to C, where a small decrease in reduction rates was calculated. In period D, however, the reduction rates continued to increase. For the larger PM10 fraction, the reduction rates were also slightly higher than for PM1.

**Table 1:** Arithmetic means and standard deviation (particles/m^3^) in front of and behind the filter unit for the two particle fractions PM1 (particles between 0.3 and 1 μm) and PM10 (particles between 0.3 and 10 μm) for all four fattening periods (A-D) and over the entire study period.

Period and particle class	Arithmetic mean in front of the filter unit	Standard deviation in front of the filter unit	Arithmetic mean behind the filter unit	Standard deviation behind the filter unit	Reduction rate [%]
A PM1	105,587,855.8	8,022,048.36	8,022,048.4	9,768,026.6	92.40
A PM10	108,019,155.6	93,015,360.97	8,106,036.0	9,830,473.9	92.50
B PM1	43,436,350	47,095,058.53	2,787,200	2,998,097.0	93.58
B PM10	45,110,312.5	47,329,719.24	2,812,429.2	2,986,989.7	93.77
C PM1	18,368,670.8	9,714,705.74	1,185,166.7	885,922.8	93.55
C PM10	19,662,345.8	10,261,374.2	1,194,229.2	885,850.0	93.93
D PM1	89,533,920.8	89,937,343.4	3,198,191.7	3,536,939.8	96.43
D PM10	90,764,950	90,723,117.1	3,211,583.3	3,543,454.4	96.46
All PM1	62,934,321.0	76,471,565.9	3,666,112.4	5,788,156.9	94.17
All PM10	64,567,537.6	76,866,532.7	3,697,433.7	5,823,668.3	94.27

The corresponding reduction rates are given in percentage.

**Table 2:** Arithmetic means (particles/cm^3^) in front of and behind the filter unit and their standard deviation from nanoparticle counter (particles between 0.02 and 1 μm) for all four periods (A-D) and over the entire study period.

Period	Arithmetic mean in front of the filter unit	Standard deviation in front of the filter unit	Arithmetic mean behind the filter unit	Standard deviation behind the filter unit	Reduction rate [%]
A	5,369.14	1,658.02	357.09	267.51	93.35
B	5,083.87	2,302.36	260.51	312.73	94.88
C	4,745.62	2,508.48	149.41	313.76	96.85
D	4,733.07	3,017.26	103.33	77.64	97.82
Total	4,963.49	2,466.44	208.62	279.81	95.80

The corresponding reduction rates are given in percentage.

The results of the nanoparticle counter referred to particles with a size between 0.02 and 1 μm. The arithmetic mean values in front of and behind the filter unit were 4963.49 and 208.62, respectively (Table 2). The calculated reduction efficiencies also increased from period to period and were between 93.35 and 97.82%. Across all periods, the value over the entire study was 95.80% (see Table 2). The daily averages in front of the filter unit ranged from 1,445.52 to 11,267.19 particles per cm^3^ of air. Behind the filter unit, the values at the measurement days ranged from 19.79 to 844.35 particles per cm^3^ of air.

### 3.2 Microbiology analysis

The microbiological results were divided into three microorganism groups, and the values in Figure 2 were logarithmized and given in CFU per m^3^ of air. The highest germ concentration was always found in the barn air. Results for the TBC indicated that even unfiltered air would have had a negligible effect on the concentration in the barn. For molds, unfiltered air would have been expected to have an effect. Yeast would have had a small effect. However, a clear reduction from in front of to behind of the filter unit was found for all types of germs. From these differences, reduction rates were calculated and are shown in Tables 3–5.

**Table 3:** Total bacteria count (TBC) concentration (colony-forming units/m^3^) given as median and interquartile range (IQR) in front of and behind the filter unit for each period and the entire study.

Period	In front of the filter unit Median (IQR)	Behind the filter unit Median (IQR)	Median reduction rate [%]
A	10,705 (5,242–14,934)	717 (123–1,334)	97.93
B	913 (336–2,557)	24 (13–69)	95.84
C	2,580 (850–7,098)	96 (25–492)	97.32
D	3,239 (1,232-12,787)	290 (15–1,003)	95.16
Total	3,235 (955–10,449)	85 (18–702)	95.88

The reduction rates (calculated from the daily arithmetic mean) are presented as percentages.

**Table 4:** Mold concentrations (colony-forming units/m^3^) given as median and interquartile range (IQR) in front of and behind the filter unit for each period and the entire study.

Period	In front of the filter unit Median (IQR)	Behind the filter unit Median (IQR)	Median reduction rate [%]
A	258 (94–535)	24 (8–62)	74.04
B	460 (240–905)	8 (8–14)	98.04
C	742 (330–1,168)	15 (12–20)	97.10
D	133 (96–193)	9 (8–10)	93.31
Total	295 (133–728)	9 (8–20)	94.64

The reduction rates (calculated from the daily arithmetic mean) are presented as percentages.

**Table 5:** Yeast concentrations (colony-forming units/m^3^) given as median and interquartile range (IQR) in front of and behind the filter unit for each period and the entire study.

Period	In front of the filter unit Median (IQR)	Behind the filter unit Median AF	Median reduction rate [%]
A	39 (21–172)	9 (8–13)	72.86
B	11 (9–34)	8 (8–9)	28.78
C	30 (19–60)	8 (8–8)	67.71
D	24 (20–37)	9 (8–9)	65.62
Total	23 (13–52)	8 (8–9)	66.03

The reduction rates (calculated from the daily arithmetic mean) are presented as percentages.

### 3.3 Statistics

None of the measured microbial counts showed a normal distribution. The pairwise comparisons between concentrations in front of and behind the filter unit revealed highly significant differences for all microbiological parameters (*p* < 0.0001, Wilcoxon Rank Sum Test).

The Kendall’s Tau-b correlation coefficient was determined to analyze the dependency of the individual parameters (Table 6). For all parameters, there was a correlation from in front of to behind filter unit, except for molds and yeasts. There was also a correlation between PM1 and PM10 in front of the filter unit. Another correlation was observed between PM10 particles and TBC in the outer air. No correlation was found between the initial concentration PM1 and the nanoparticles.

**Table 6:** Significant Kendall’s Tau-b correlation coefficients divided into the locations in front of and behind the filter unit.

Variable 1	Location 1	Variable 2	Location2	*p*-value	Kendall‘s Tau
nanoparticle	in front of	nanoparticle	behind	0.0006	0.44368
PM1	in front of	PM1	behind	<0.0001	0.80690
PM1	in front of	PM10	behind	<0.0001	0.80230
PM10	in front of	PM10	behind	<0.0001	0.78851
PM10	in front of	PM1	in front of	<0.0001	0.97701
PM10	in front of	TBC	in front of	0.0438	0.25977
PM10	in front of	PM1	behind	<0.0001	0.79310
TBC	behind	TBC	in front of	<0.0001	0.50968
TBC	behind	PM10	in front of	0.0145	0.31494
TBC	behind	PM1	in front of	0.0160	0.31034
yeasts	in front of	TBC	in front of	0.0019	0.39355
yeasts	in front of	molds	in front of	0.0283	0.27742
yeasts	in front of	PM1	behind	0.0477	0.25517
yeasts	in front of	TBC	behind	0.0103	0.32473
PM1	behind	nanoparticle	behind	0.0477	0.25517
PM1	behind	PM10	behind	<0.0001	0.97701
yeasts	behind	molds	behind	0.0158	0.30637

The correlations were tested for the variables nanoparticles, PM1, PM10, total bacteria count (TBC), molds, and yeasts.

### 3.4 PCR

The results from the Coriolis air sampler showed no influenza A RNA copies. On January 17, 2023 (A3), three positive TRT samples were found in the air of the barn. More positive results were found for ORT. Positive results were obtained with the Coriolis air sampler in front of the filter on five of seven days in the first period. There were no positive results behind the filter unit. On two measurement days, there was also a positive result in the barn during the first period. In the second fattening period, there was one positive result in front of the filter unit on one day. Again, no DNA from ORT was found behind the filter with the Coriolis air sampler (Table 7). The comparison of 15 samples in front of and behind the filter unit with six positive samples in front of the filter unit versus no detection behind it was significantly different when conducting the Fisher’s exact test (*p* = 0.0105).

**Table 7:** Results of PCR analysis from influenza A, *Turkey Rhinotreachetis virus* (TRT), *Ornithobacterium rhinotracheale* (ORT).

Period	Coriolis in front of the filter unit	Coriolis behind the filter unit	Coriolis barn	Teflon in front of the filter	Teflon behind the filter	PC-filter in front of the filter unit	PC-filter behind the filter unit	Coarse dust filter
Influenza A	ND	ND	ND	1	ND	2	ND	ND
TRT	ND	ND	1	ND	ND	/	/	ND
ORT	6	ND	2	13	1	/	/	3
Total	15	15	15	20	20	3	3	4

The results are divided into the values from the Coriolis air sampler, the stationary pump with Teflon and PC-Filter, and from the dust from the coarse dust filter. If no positive results were reported by the laboratory, not detectable (ND) was indicated.

From the beginning of the second period (B), the results showed a positive influenza A result in front of the filter unit in a measurement period with a Ct value of 35 collected with a Teflon filter. TRT RNA copies could not be detected with this measurement technique in any period. The results for ORT were more comprehensive. A total of 13 of 20 measurement periods between the measurement days showed a positive result in front of the filter unit with Ct values between 26 and 34. ORT DNA was found once behind the filter unit in the interval between the first and second measurement day; however, with a significantly higher Ct value of 38. During the measurements with the PC filters, two positive influenza findings with CT values between 32.2 and 36.6 were found in two samples in the outside air during the acute outbreak. Sequencing of the positive influenza findings only worked for the PC filter samples. Sequencing detected H9 Influenza A gene fragments in both PC filter samples. Positive ORT results were detectable for the dust from the coarse dust filters in three filter modules.

### 3.5 Barn climate parameters

The results of the hand-held instruments are shown in Table 8. The arithmetic means, the standard deviations, maxima, and minima are displayed for all parameters measured at nine locations.

**Table 8:** Barn climate parameters measured with hand-held instruments in an area network measurement.

	Temperature [°C]	Relative humidity [%]	Air velocity [m/s]	NH_3_ [ppm]	CO_2_ [ppm]	Light intensity [lx]
Arithmetic mean	19.39	61.66	0.17	6.18	1,335.56	147.28
Standard deviation	3.54	6.94	0.14	4.54	419.20	229.66
Maximum	27.40	75.20	0.82	18.00	2,700.00	2,442.33
Minimum	13.50	41.30	0.00	0.00	700.00	19.33

The arithmetic mean, the standard deviation, the maximum and the minimum for temperature [°C], relative humidity [%], air velocity [m/s], NH_3_ [ppm], CO_2_ [ppm], and light intensity [lx] over all measurement days (*n* = 31) are shown.

**Figure 3:**
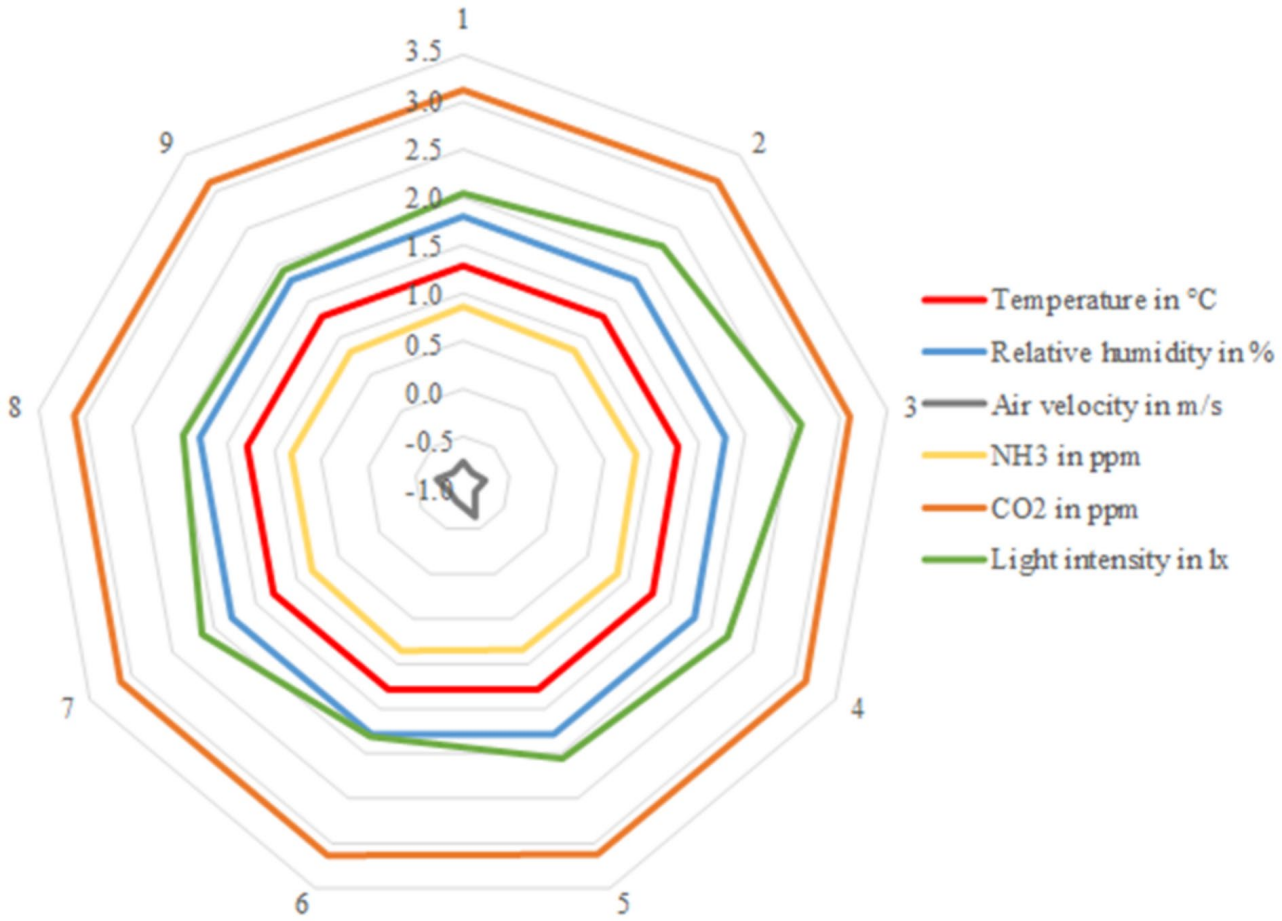
Logarithmized mean values from the nine different sampling points in the barn. Displayed climate parameters are: temperature in °C, relative humidity in %, air velocity in m/s, NH_3_ in ppm, CO_2_ in ppm. Averages from *n* = 31 samples at each sampling point.

In Figure 3 it can be seen clearly that the values for all barn climate parameters were similar at the different measurement points, with the exceptions of light intensity and air velocity.

## 4 Discussion

In the presented study, a naturally ventilated turkey barn was equipped with filter modules that enabled a switch from a naturally to a mechanically ventilated barn. The aim of the investigations was to test the effectiveness of such filter systems in an agricultural environment. A further goal was to assess whether such a retrofit solution can contribute to an increased biosecurity when naturally ventilated barns are converted to a mechanically ventilated system with filter units. The filter efficiency of the modules concerning particles and germs were investigated over the course of approximately 16 months. Additionally, climate parameters were measured at farm visits to estimate the air dispersion in the barn.

Suspended bioaerosols are airborne particles of different sizes. Therefore, measuring particle classes can help to assess the reduction efficiency of filters. However, bioaerosols may have characteristics such as viability, infectivity, or other adverse health effects in which they differ from other particles. Therefore, the investigation of both particles and viable bioaerosols contribute to estimating the usefulness of filters. When looking at particle concentrations PM1, PM10, and nanoparticles, a clear reduction by the filter unit was observed (Tables 1, 2). Changes in efficiency were also detected over the course of the experimental phase, and it seems that the filter efficiency increased during the course of the study. This increase in filtration efficiency is perhaps related to effects on the filter. For instance, the dust deposited on the filter over time makes it more difficult for other dust particles to pass through the filter. However, there was a slight decrease in filter performance for PM1 from period B to C, which can be explained by fluctuations. By assuming that most of the viable and potential infectious microorganisms are bound to particles (5, 16, 21), this means that the probability of airborne contamination can be significantly reduced or even prevented. Although the calculated reduction rates are not 100%, the reduction achieved by the combination of coarse and fine dust filters is highly significant. Considering that many cultivable bacteria are expected, especially in the large size class like PM10, it can be hypothesized that potential harmful microorganisms and viruses, including non-culturable infectious particles, are reduced by more than Log_10_ (6).

To the best of our knowledge, this was the first time that such a study was investigated under practical conditions in an agricultural environment. The coarse dust filters had to be exchanged after the second period because of the high pressure loss due to continuous operation and the prevailing dust load. However, the test phase of the system was terminated as planned after the fourth period (D). Thus, it can be concluded that the fine dust filters can be operated for at least 16 months, probably even longer. It is known from other studies in other environments that coarse filters can help filter fine dust and thus protect against a drop in pressure in the fine dust filters (22). Course filters are far less expensive and therefore more economical. Furthermore, if the system is operated in such a way, that it is only activated in acute risk situations, the operating time would be extended accordingly. In this project, the total costs for the system and its operating costs were not estimated. The aim was first to investigate its efficacy and to assess its effects on the barn’s climate. The cost effectiveness needs to be evaluated in future by considering the health and performance of animals and the potential risk reduction. Poultry producers have lost millions of birds and some of them also their economic existence due to highly pathogenic AI outbreaks (23).

For the microbiological results for TBC and molds, with the exception of period A (74.04%) for molds, in all four periods (A-D) filters retained over 93% of the respective germs (Tables 3, 4). The filtration efficiency for yeasts were much lower in the individual periods. However, this can be partly explained by the fact that the initial concentrations in front of the filter unit were already low and the detection limit was used as a theoretical value for the values behind the filter unit. For example, the initial concentration of period B for yeasts was very low, with a median of 11 colony-forming units per m^3^ of air (Table 5). Deviations at counting low concentrations could have had a stronger impact on the results.

The efficient filter performance was obvious in the plots (Figure 2) and statistically confirmed by the Wilcoxon Rank Sum Test. A significant difference between in front of and behind the filter unit was calculated for all parameters (*p* < 0.0001). Regarding the correlations between the parameters, it was expected that the concentrations in front of and behind the filter unit would show a dependence. For the microbiological parameters, this was only the case for TBC, that showed a high correlation. For molds and yeasts, it should be noted that the initial concentration for these two types of germs was relatively low, and the theoretical detection limit was often used for the post-filter values. This could be a possible explanation for non-correlating concentrations. The particle fractions PM1 and PM10 correlated highly. It is important to note that these fractions were not particles with a diameter of 1 or 10 μm, but rather summed particles with diameters up to 1 or 10 μm. This means that all particles included in PM1 automatically fall into the PM10 category. The calculated correlation between PM10 and outdoor TBC supports the theory that bacteria are primarily attached to larger particles, although they formed only a small fraction (approx. 0.8% of the particles were between 3 and 20 μm in this investigation), which could be the reason for a more moderate correlation. The particles measured for PM1 and the particles measured for the nanoparticles were considered up to a size of 1 μm. However, there was no correlation between the two parameters in the outdoor air. The reason may be that the aerosol spectrometer only detects particles as small as 0.3 μm, while the nanoparticle counter can detect even smaller particles as small as 0.02 μm. Particles below 0.3 μm showed higher concentrations of approximately factor 779 [(nanoparticle concentration-PM1)/PM1; Tables 1, 2].

The measurements with the nanoparticle counter and the Coriolis air sampler had to be taken with over half an hour time delay in front of and behind filter unit because only one device was available. This may have slightly affected the comparability, but the significant reduction in concentrations was mainly due to the filter performance and not to the time delay because the results were confirmed again through frequent repetition. In addition, these results are consistent with those obtained using the aerosol spectrometer, where two devices were operated in parallel.

In order to detect nucleic acids in the air, the appropriate measurement technology must be used (24). Each microorganism and virus has its own characteristics and behaves differently when aerosolized under different conditions (21). Nevertheless, influenza A virus RNA could be detected once in a Teflon filter and twice in PC filters (influenza A-H9) in the unfiltered air. No virus was found behind the filter unit during the respective test periods. Unfortunately, the DNA of the Teflon filter could not have been sequenced due to too little material (high CT value). The results give only a first indication that the filter unit can retain pathogenic viruses. However, these three results are too few for statistical analyses. To investigate the reduction efficiency systematically, the used filters or parts of the used filters could, for example, be integrated in bioaerosol test ducts in future studies. This would enable reproducible results to be generated. The ORT results were much more comprehensive. With the Coriolis air sampler, DNA was found in front of the filter unit at 6 of the 15 examined measurement days. Behind the filter unit, all results were negative. This difference was significant (*p* = 0.0105) and shows that pathogenic bacteria can be retained by the filter modules, as it can be assumed that the detected DNA was part of bacterial cells or cell fragments. On two days, the Coriolis air sampler in the barn was also positive for ORT. Whether the bacteria were from birds in the barn or entered the barn by other transmission sources remains unknown. By using Teflon filters with stationary pumps, it was also shown that the ambient pressure of ORT was high during the study period, as 13 of 20 measurement periods showed positive results in the outdoor air, whereas the filtered air remained negative in 95% of the cases. In addition, three of four coarse filters from the filter modules had positive ORT dust samples. Therefore, the ambient air contamination with ORT appears to have been high. The source remained unclear. However, the filter unit may not be completely impenetrable to bacteria, as there was also a positive result in the filtered supply air in one measurement period. Nonetheless, the PCR showed a higher Ct value. While the results in front of the filter had CT values between 26 and 34, the result behind the filter unit had a higher value of 38, indicating that a small amount of DNA was detected behind the filter. TRT was only once detected within the barn. If animals were infected remains unknown. The result could also be a consequence of vaccination with live vaccines (25). However, the virus was not detected outside and therefore no reduction could have been observed for this indicator aerosol.

The system change from a naturally ventilated barn to a quasi-mechanically ventilated barn was carried out in such a way that there were no observed negative effects on the animal health and the climate parameters in the barn. Although there was no direct reference barn against which the barn climate values could be compared, the measured barn climate parameters indicated good air quality and air distribution inside the barn.

The laws applicable in Germany were complied with in the study. The maximum values for gases of 3,000 ppm CO_2_ and 20 ppm NH_3_ are prescribed in the specific laws for keeping turkeys (26). These gas concentrations were not reached in the tests. The light intensity was below the prescribed 20 lx at two measuring points on the very last measuring day. This may be due to the fact that the curtains were dusty, and it was a very cloudy day. However, when installing such a system, care must be taken to ensure that the animals continue to have sufficient light available. The average air velocity also shows that the system does not create drafts at animal level.

Nonetheless, the barn climate parameters were only measured in the morning, so the results are only snapshots. During the night, temperature and gas concentrations may have been different due to low animal activity and lower airflow (as ventilation is temperature controlled) (27). Whether this would have changed the air distribution remains unclear and should be the subject of future studies. However, the results were obtained over a long period of time, including situations with low air exchange rates (e.g., young animals in winter) and periods with high air exchange rates (final fattening phase in summer), and no significant differences in the parameters were found between the measurement points. The system therefore appears to be able to provide good air distribution in this barn on a permanent basis. It should be noted that the barn climate results can certainly be influenced by barn type, barn size, and barn dimensions, and it would therefore be useful to test the system in other barns to see whether similarly good air quality and air distribution are possible in larger barns with different dimensions.

Management could be a critical factor in successful livestock production. However, new systems can make successful management much easier. Further development of housing systems can have a positive impact on animal welfare, but also on biosecurity and therefore animal health. The controlled supply of filtered air can be an important component in preventing the entry of pathogens. Especially in the case of AI, an alternative to the current approach may contribute to biosecurity. Infected animals must currently be culled and eliminated (12). Whether it is through the introduction of vaccination or increased biosecurity, or a combination of both, the prospect of avoiding mass culling should be promoted as soon as possible. The importance of airborne entry of pathogens into livestock farms is currently being discussed. There have been many discussions concerning AI in particular (8, 15). Nevertheless, the tested system offers for the first time the possibility to prevent or reduce the uncontrolled airborne entry of important pathogens such as influenza viruses, ORT, *Mycoplasma* etc. as a retrofit solution (2, 28, 29). This additional technology can enhance the biosecurity of susceptible farm animals but only if all other obligatory and necessary biosecurity measures are carefully implemented.

To conclude, adapting filter modules with fine dust filters at poultry barns is suitable to reduce efficiently particle fractions, which act as main carriers for airborne pathogens in nature. This additional biosecurity measure can help to protect flocks from an uncontrollable entry via air, especially in regions with higher risks pathogen transmissions. However, changes of the ventilation systems should not decline the barn climate and the air quality.

## Data Availability

The raw data supporting the conclusions of this article will be made available by the authors, without undue reservation.
